# Association of Parasite Biomass with Disease Severity in *Plasmodium vivax* and *Plasmodium falciparum* Malaria Infections

**DOI:** 10.4269/ajtmh.26-0106

**Published:** 2026-08-27

**Authors:** Prasad H. Babar, Jacob A. Tennessen, Rimi Chakrabarti, Jayashri T. Walke, Usheer Kanjee, Anvily Almeida, Mezia Fernandes, Pooja Gawas, Laura Karschney, John White, Marina Vaz, Edwin Gomes, Pradipsinh K. Rathod, Manoj T. Duraisingh

**Affiliations:** ^1^ Department of Chemistry, University of Washington, Seattle, Washington, USA;; ^2^ Department of Medicine, Goa Medical College and Hospital, Bambolim, India;; ^3^ Department of Immunology and Infectious Diseases, Harvard T.H. Chan School of Public Health, Boston, Massachusetts, USA

## Abstract

The clinical and parasitological factors underlying severe malaria pathogenesis remain incompletely understood, particularly in *Plasmodium vivax* (*P. vivax*). To address this issue, clinical and parasitological metrics, including circulating peripheral parasitemia and the parasite biomass (the total number of parasites in the body consisting of both circulating peripheral and noncirculating parasites), were measured in patients with severe and uncomplicated monoinfections of *P. vivax* and *Plasmodium falciparum* (*P. falciparum*) malaria who presented at a hospital in southwest India. Peripheral parasitemia was positively correlated with disease severity in *P. falciparum* but negatively correlated with disease severity in *P. vivax.* Contrarily, the parasite biomass, estimated by the levels of circulating antigens *P. vivax* lactate dehydrogenase and *P. falciparum* histidine-rich protein 2 for *P. vivax* and *P. falciparum*, respectively, was positively correlated with both the degree of severity and the length of hospital stay in both *P. vivax* and *P. falciparum* cases. Together, these observations indicate a significant hidden parasite biomass in *P. vivax* infections and suggest that parasite biomass is a more robust parasitological marker of severity than parasitemia. Notably, thrombocytopenia was also positively correlated with parasite biomass and longer hospital stay in both *P. vivax* and *P. falciparum* infections. These results highlight the importance of parasite biomass as a leading factor in the disease severity of malaria infections in India.

## INTRODUCTION

In 2024, there were 282 million estimated malaria cases globally. Although the vast majority of the 610,000 malaria-related deaths were caused by *Plasmodium falciparum* (*P. falciparum*), *Plasmodium vivax* (*P. vivax*) has been the most geographically widespread of the malaria parasites.^1^ *Plasmodium vivax* infections were historically considered benign, although there is a steady increase in evidence of severe and fatal *P. vivax* infections.^2^^,^^3^ Unlike *P. falciparum*, in which parasite sequestration in tissue microvasculature is key for severe illness,^4^ the disease progression to severity in *P. vivax* malaria is not clearly understood.^5^^,^^6^ *P. vivax* parasite invasion is restricted to reticulocytes; thus, peripheral parasitemia does not reach the levels commonly seen in *P. falciparum* infections.^7^^,^^8^ Additionally, parasite cytoadherence in severe *P. vivax* malaria is incompletely understood compared with *P. falciparum* malaria.^3^^,^^6^ There is evidence of possible parasite accumulation in *P. vivax* infections, given that mature stages of *P. vivax* parasites were less frequently found in peripheral blood smears^7^ and in vitro selective sequestration studies revealed that the *P. vivax* parasites bind to lung, placenta, and brain cells, although at a much lower level compared with *P. falciparum.*^9^^,^^10^ Further parasite biomass estimation using *P. vivax* lactate dehydrogenase (PvLDH), a serum circulating antigen, as a marker has revealed that parasite biomass is higher in severe cases than uncomplicated ones.^11^^,^^12^ Significant levels of parasite accumulation in the bone marrow and spleen in acute and chronic *P. vivax* infections, often in the absence of corresponding peripheral parasitemia, strongly suggested enrichment of infected erythrocytes in these niches.^13^^,^^14^ Like *P. falciparum*, a marked increase in inflammatory cytokines and higher parasite burden can also be important factors in severe *P. vivax* infections.^15^

Interestingly, thrombocytopenia, which is not yet considered a severity criterion by the WHO, has been a common clinical factor associated with severe *P. vivax* and *P. falciparum* cases.^16^^–^^19^ The magnitude of thrombocytopenia and mortality risks associated with it might vary for both *P. vivax* and *P. falciparum* infections,^17^^,^^19^^,^^20^ and the mechanism of developing thrombocytopenia in severe cases is not clear, although binding of platelets directly to parasites and their phagocytosis might be probable reasons.^21^^,^^22^

Here, a retrospective hospital-based study of malaria patients enrolled by the South Asia International Center of Excellence for Malaria Research (MESA-ICEMR) in Goa, India, is described with the aim of understanding parasitological and clinical factors contributing to disease severity in *P. vivax* and *P. falciparum* malaria. This low-malaria transmission region experiences transmission throughout the year and is coendemic for both *P. falciparum* and *P. vivax.*^23^^,^^24^ Considering that parasite biomass in *P. vivax* pathophysiology seems to play a vital role^15^ and that the synergistic diagnostic value of parasite biomass with thrombocytopenia in severe malaria could be vital for clinical management of severe malaria,^25^ multiple clinical and parasitological parameters were measured, including parasitemia, parasite biomass (through plasma PfHRP2 and PvLDH levels) and platelet counts to probe whether these factors are associated with disease severity in severe malaria patients. Malaria patients were classified as severe if they met at least one of the severity criteria as defined by the WHO.^26^ The authors further investigated the association of these parameters with the length of hospital stay in *P. vivax* and *P. falciparum* patients. A positive association between parasite biomass and disease severity in both *P. vivax* and *P. falciparum* severe cases was observed in the present study. The discordance between parasitemia and disease severity further suggested parasite accumulation in severe *P. vivax* cases. Positive correlations between thrombocytopenia and the disease outcomes of severity and hospital stay duration are also reported. The present study indicates that parasite biomass and thrombocytopenia might be predictive of severity and length of hospital stay in severe malaria infections, signifying the use of these factors in effective clinical management of malaria.

## MATERIALS AND METHODS

### Study population.

Care-seeking febrile individuals advised for a malaria smear test at Goa Medical College (GMC) were screened (Supplemental Figure 1). Malaria-positive patients aged 1 to 65 years were enrolled, excluding pregnant individuals and those suffering from chronic diseases. A 6-mL venous blood draw was collected from consenting patients. Various clinical point-of-care parameters were measured for inpatients. Patients were classified as having severe or uncomplicated malaria according to WHO guidelines, with slight modifications detailed below.^26^ Severe malaria in malaria-positive patients was defined as the patients having at least one of the following symptoms: 1) impaired consciousness: Glasgow Coma Score <11 in adults (>12 years) or Blantyre Coma Score <3 in children (<12 years), 2) acidosis: plasma bicarbonate <15 mmol/L, 3) hypoglycemia: blood or plasma glucose <40 mg/dL, 4) severe malarial anemia: hemoglobin (Hb) concentration <7 g/dL or a hematocrit (HCT) level of <20% in adults or Hb concentration <5 g/dL or an HCT level of <15% in children, together with parasite positivity, which is a slightly more lenient than the WHO criteria of 10,000 parasites/*µ*L, 5) renal impairment: plasma or serum creatinine >3 mg/dL or blood urea >20 mmol/L (48 mg/dL), 6) jaundice: plasma or serum bilirubin >3 mg/dL together with parasite positivity, which is a slightly more lenient than the WHO criterion of 10,000 parasites/*µ*L, 7) pulmonary edema: when it was radiologically confirmed by GMC, 8) respiratory distress, which was considered a separate criterion rather than a part of the WHO’s definition of pulmonary edema: respiratory rate >20 breaths/minute or partial pressure of oxygen in the alveoli of <75 mm Hg,^27^^,^^28^ 9) significant bleeding: including recurrent or prolonged bleeding from the nose, gums, or venipuncture sites, 10) shock: systolic blood pressure (BP) <80 mm Hg in adults or <70 mm Hg in children, or 11) hyperparasitemia: *P. falciparum* parasitemia >10%. For outpatients, Hb and HCT levels at enrollment were considered. The number of severity criteria met by a malaria-positive patient was designated as the degree of severity. For inpatients, the lowest Hb and HCT levels over the entire length of hospitalization were considered.^28^^,^^29^ All the infection-related parameters, including parasitemia, parasite density, gametocytemia, and gametocyte density, for each patient were assessed by a trained microscopist at the MESA-ICEMR laboratory at enrollment. Platelet counts were rounded off to the nearest thousand. A total of 1,219 *P. vivax* cases (1,042 uncomplicated, 177 severe) and 482 *P. falciparum* cases (285 uncomplicated, 197 severe) were assessed for disease severity indicators (whole dataset), and the patients were enrolled at GMC (2012–2023). Severe malaria patients were not routinely monitored with a lumbar puncture on admission to GMC; however, the patients were continuously monitored for any comorbidities during their stay at the hospital. The PfHRP2 and PvLDH levels in a random subset of 273 *P. vivax* (176 uncomplicated, 101 severe) and 113 *P. falciparum* malaria patients (113 uncomplicated, 78 severe) were respectively measured to estimate parasite biomass (Parasite Biomass subset). The parasite biomass of only a subset of the total enrolled samples could be assessed. A proportion of the enrolled patients self-reported pretreatment, and their samples were included in the analysis as “treated” samples, whereas samples from patients with no pretreatment at the time of enrollment were categorized as “untreated.”

### ELISA.

For the PvLDH ELISA, Nunc Maxisorp plates (Thermo Fisher Scientific [Waltham, MA, USA]) were coated overnight at 4°C with 50 *µ*L/well capture antibody (2.5 *µ*g/mL, CABT-B8661; Creative Diagnostics [Shirley, NY, USA]) in phosphate-buffered saline (PBS) at 4°C overnight in a humid chamber. Subsequent incubations were performed at room temperature. After blocking with 100 *µ*L of 1% (weight per volume) bovine serum albumin (BSA)/PBS for 1 hour, 50 *µ*L of standards and samples (1:2 to 1:20 dilution in PBS/Tween at desired detectable dilution (1:2 to 1:20) with 1 hour of incubation. Standards were prepared similarly using PvLDH recombinant protein (range: 60 ng/mL to 0.93 ng/mL). A total of 50 *µ*L of PvLDH detection antibody (biotinylated at 1.25 *µ*g/mL in PBS/Tween, CABT-B8662; Creative Diagnostics) was then incubated for 4 hours, followed by the addition of 50 *µ*L of horseradish peroxidase-Streptavidin (1:10,000 dilution in 1% w/v BSA/PBS). The mixture was then incubated for 1 hour. After each step, the wells were washed four times with 0.05% (v/v) PBS/Tween. 3,3′,5,5′-tetramethylbenzidine substrate (50 *µ*L, T0440; Sigma-Aldrich [St. Louis, MO, USA]) was added and left for 30 minutes; the reaction was stopped using 50 *µ*L of 1 M hydrochloric acid, and absorbance was measured at 450 nm. PfHRP2 ELISA was performed using a previously established protocol.^27^^,^^28^

## STATISTICAL ANALYSES

Variables with skewed distributions were log-transformed, adding a constant to all values when needed to facilitate log transformation. The Mann–Whitney test was used for bivariate comparisons. Spearman correlation analysis was used to quantify the association between two variables, and a simple linear regression analysis was used to estimate the best fit for the relationship between these variables. LASSO linear models were generated using glmnet^30^ in R (v. 4.3.3; R Foundation [Vienna, Austria]) to identify plausible multivariate models for predicting malaria severity and days in the hospital. The two discrete integer response variables (severity and days) were log-transformed (base e) and modeled as continuous in linear regression; severity was also modeled as binary (severe versus uncomplicated) in logistic regression. Only inpatient data were included, and missing data were imputed using the mean for each variable. For malaria severity, only potential predictors that had not been used directly to determine severity were included, such as parasitemia, parasite density, gametocytemia, gametocyte density, parasite biomass (measured as PvLDH or PfHRP2), platelets, age, and sex. For days in the hospital, all parameters, including other measured clinical variables that were not disease outcomes, were included. Because many patient samples had missing PvLDH and PfHRP2 data, models were run both excluding these values as a potential factor and including them, but only considering patient samples with non-missing biomass data. These latter biomass models had smaller sample sizes, so they are included to explore the potential impact of biomass, whereas the full effects of other variables are best represented by the larger datasets that ignore biomass. For all multivariate analyses, increasingly complex models were assessed, starting with the best explanatory parameter in simple linear regression and iteratively adding optimal explanatory variables as selected by glmnet. For a given lambda value, glmnet identifies the set of explanatory variables that best predict the response. The model with the lowest lambda (largest number of coefficients) for which all variables had uncorrected *P* <0.05 in multiple regression was chosen. Pretreatment was included as a binary variable in all models, regardless of its significance, to account for this important cofactor. To further assess the notable contrasting effects of parasitemia and biomass on vivax severity, a logistic regression model was fit using all vivax patients for whom both of these parameters had been measured: log (odds of severe malaria) ∼ log (PvLDH) + log (parasitemia + 0.01). The minimum effect size was estimated to gauge whether the dataset had the statistical power to significantly distinguish the difference in parasite biomass between uncomplicated and severe infections. For various hypothetical sample sizes, the minimum effect size that would yield a significant difference between severe and uncomplicated samples, given the empirical variance, was estimated. For *P. vivax*, only the number of severe cases was varied, and the number of uncomplicated cases was maintained at the empirical value of 171. For *P. falciparum*, the sample sizes of both severe and uncomplicated cases were varied, keeping them equal. The study dataset had the statistical power to detect a 1.6-fold difference for PvLDH and a 2.4-fold difference for PfHRP2.

## RESULTS

### *Plasmodium vivax* infections exhibit less severe clinical phenotypes than *P. falciparum* infections.

The authors of the present study wanted to investigate the associations between various parasitological parameters and clinical phenotypes of *P. vivax* and *P. falciparum* infections. To achieve this, they first assessed various clinical parameters (Table 1) and clinical outcomes (Table 2) for uncomplicated and severe infections with *P. vivax* and *P. falciparum*. The median ages for severe and uncomplicated *P. vivax* were 25 and 27 years, respectively, and for *P. falciparum*, they were 25 and 26 years, respectively. Most of the enrolled study participants were male, comprising 91% of the *P. vivax*-infected patients and 89% of the *P. falciparum*-infected patients. Uncomplicated infections comprised 86% of *P. vivax* infections and 59% of *P. falciparum* infections. Multiple clinical parameters were significantly different between uncomplicated and severe malaria patients for both species, including days in hospital, Hb, HCT, respiration rate, temperature at enrollment, blood urea nitrogen, and bilirubin levels. Plasma bicarbonate levels were only significantly different between severe and uncomplicated *P. vivax* cases, whereas other clinical parameters, such as platelet, systolic BP, and serum creatinine levels, were only significantly different between severe and uncomplicated *P. falciparum* cases. Systolic BP, plasma bicarbonate, blood urea nitrogen, serum creatinine, and the highest total bilirubin levels were significantly different between severe *P. vivax* and *P. falciparum* cases, as seen in previous studies.^31^^,^^32^

**Table 1:** Comparison between clinical point of care parameter values measured for uncomplicated and severe *Plasmodium vivax* and *Plasmodium falciparum* patients in the untreated and treated dataset

Parameter	Uncomplicated Pv, Median (IQR; *n*)	Severe Pv, Median (IQR; *n*)	*P*-Value	Uncomplicated Pf, Median (IQR; *n*)	Severe Pf, Median (IQR; *n*)	*P*-Value	Severe Pv/Pf *P*-Value
Parasite-related							
Parasitemia (%)	0.17 (0.05–0.6; 1,039)	0.03 (0–0.13; 170)	<0.0001	0.34 (0.10–0.94; 284)	0.25 (0–1.9; 197)	0.36	<0.0001
Parasite density (parasites/*µ*L)	1,965 (560–5,920; 1,036)	860 (0–4,066; 174)	<0.0001	2,905 (618.5–9,920; 281)	650 (0–7,238; 196)	0.0003	0.2704
Parasite biomass* (ng/*µ*L)	23.03 (10.7–68.9; 173)	55.2 (17.1–178; 100)	0.0001	3.18 (1–10.4; 39)	7.55 (2.5–38.2; 74)	0.0165	<0.0001
Clinical							
Days in hospital	0 (0–0; 1,038)	4 (1–6; 177)	<0.0001	0 (0–0; 284)	5 (3–8; 197)	<0.0001	<0.0001
Hemoglobin (g/dL)	11.9 (10.6–12.98; 1,020)	9.9 (7.85–11.5; 173)	<0.0001	11.4 (10.1–12.6; 263)	9.05 (7.2–11.1; 176)	<0.0001	0.0989
Hematocrit (%)	36 (31–41; 986)	28 (19–36) [167]	<0.0001	35.5 (30–39.2; 230)	28 (21.7–35; 162)	<0.0001	0.6216
Platelets/*µ*L*	8 (6–14 × 10^4^; 55)	8 (4–14.25 × 10^4^; 130)	0.4041	10.5 (6–15 × 10^4^; 64)	7 (4–15 × 10^4^; 179)	0.0195	0.9541
Systolic blood pressure (mm Hg)	100 (90–109.5; 56)	100 (85–106; 133)	0.0961	100 (100–110; 64)	100 (90–106; 183)	0.0172	0.0445
Blood glucose (g/dL)	94 (85.5–133.8; 6)	109 (88–141; 27)	0.5923	100 (92–127; 15)	97 (85–119; 47)	0.2613	0.1911
Plasma bicarbonate (mmol/L)	22 (20.5–23.5; 9)	19 (16–21; 60)	0.0025	19 (17–21; 3)	18 (14–20; 99)	0.4682	0.0208
Respiration rate (breaths/minute)	16 (16–18; 48)	21 (18–26; 132)	<0.0001	18 (16–18; 59)	24 (18–28; 178)	<0.0001	0.0569
PaO_2_ (mm Hg)	94.8 (85.0–99.9; 8)	88 (59.2–107; 61)	0.455	85.8 (82–116.9; 3)	87.65 (67.1–105.7; 98)	0.6274	0.9093
Temperature at enrolment (°C)	100.3 (98.8–102.7; 1,040)	98.9 (97.7–100.8; 175)	<0.0001	100.3 (98.6–100.7; 283)	99.3 (98.2–101; 195)	<0.0001	0.1302
BUN* (mg/dL)	30.7 (26–39; 51)	36 (26.5–52; 125)	0.0016	26 (20.5–34; 61)	51 (32–99.5; 180)	<0.0001	<0.0001
Serum creatinine (mg/dL)	0.8 (0.7–1.1; 51)	1 (0.7–1.4; 127)	0.0653	0.8 (0.7–1; 62)	1.1 (0.8–2.1; 179)	<0.0001	0.0123
Total bilirubin (mg/dL)	1.6 (1.2–2.3; 41)	2.4 (1.1–4; 119)	0.0149	1.2 (0.6–1.7; 50)	3 (1.1–7.7; 164)	<0.0001	0.0497
Host-related							
Age	25 (20–32; 1,041)	27 (21–40; 177)	0.0006	25 (21–33; 285)	26 (20–39; 197)	0.1835	0.3885

BUN = blood urea nitrogen; IQR = interquartile range; *n* = number of patients for which the data is available; PaO_2_ = partial pressure of oxygen in alveoli; Pf = *Plasmodium falciparum*; Pv = *Plasmodium vivax*.

Median values with lower and upper confidence limits are shown. Bivariate comparison was performed using the nonparametric Mann–Whitney test. The column “Severe Pv/Pf *P*-value” contains *P*-values for the comparison of parameters between severe Pv and Pf patients. For inpatients, the lowest values of the following clinical parameters are reported here: hemoglobin, hematocrit, platelets/*µ*L, systolic blood pressure, and blood glucose. Additionally, the highest values of the following clinical parameters are reported here: plasma bicarbonate, respiration rate, PaO_2_, temperature at enrollment, BUN, serum creatinine, and total bilirubin.

* Parasite biomass: PfHRP2 level in samples from Pf-infected patients, and Pv lactate dehydrogenase level in samples from Pv-infected patients; Platelets/*µ*L are expressed as ×10^4^.

**Table 2:** Proportion of samples exhibiting clinical outcomes for uncomplicated and severe *Plasmodium vivax*- and *Plasmodium falciparum*-infected patients

Clinical Outcome	Uncomplicated *P. vivax* (*n* = 1,042)	Severe *P. vivax* (*n* = 177)	Uncomplicated *P. falciparum* (*n* = 285)	Severe *P. falciparum* (*n* = 197)
Cerebral malaria	0	8 (4.5%)	0	18 (9.1%)
Malarial anemia	0	64 (36.1%)	0	67 (34%)
Thrombocytopenia	42 (4%)	98 (55.3%)	46 (16%)	134 (68%)
Severe thrombocytopenia	8 (0.7%)	37 (20.9%)	7 (2.46%)	51 (25.8%)
Abnormal bleeding	0	3 (1.6%)	0	10 (5%)
Shock	0	14 (7.9%)	0	11 (5.5%)
Hypoglycemia	0	1 (0.5%)	0	2 (1%)
Acidosis	0	8 (4.5%)	0	30 (15.2%)
Pulmonary edema	0	15 (8.4%)	0	20 (10.1%)
Respiratory distress	0	78 (44%)	0	127 (64.4%)
Renal impairment	0	38 (21.4%)	0	96 (75.5%)
Jaundice	0	44 (24.8%)	0	80 (40.6%)
Mortality	0	2 (1.1%)	0	12 (6%)

BP = blood pressure; *P. falciparum* = *Plasmodium falciparum*; *P. vivax* = *Plasmodium vivax*; PaO_2_ = partial pressure of oxygen in alveoli.

Cerebral malaria: Glasgow Coma Score <11 in adults (>12 years) or Blantyre Coma Score <3 in children (<12 years); Malarial anemia: hemoglobin concentration <7 g/dL or a hematocrit level of <20% in adults; hemoglobin concentration <5 g/dL or a hematocrit level of <15% in children, together with parasite positivity; Thrombocytopenia: platelet count <150,000/*µ*L; Severe thrombocytopenia: platelet count <50,000/*µ*L; Abnormal bleeding: including recurrent or prolonged bleeding from the nose, gums, or venipuncture sites; Shock: systolic BP <80 mm Hg in adults or <70 mm Hg in children; Hypoglycemia: blood or plasma glucose <40 mg/dL; Acidosis: plasma bicarbonate <15 mmol/L; Pulmonary edema: radiologically confirmed; Respiratory distress: respiratory rate >20 breaths/minute or PaO_2_ <75 mm Hg with renal impairment: plasma or serum creatinine >3 mg/dL or blood urea >20 mmol/L (48 mg/dL); Jaundice: plasma or serum bilirubin >3 mg/dL, together with parasite positivity.

A species-stratified comparison of clinical outcomes among severe malaria patients revealed that shock was more prevalent in severe *P. vivax* cases, whereas cerebral malaria, thrombocytopenia, abnormal bleeding, hypoglycemia, acidosis, radiologically confirmed pulmonary edema, respiratory distress, renal impairment, jaundice, and mortality were predominant in severe *P. falciparum* cases (Table 2). Additionally, *P. vivax* patients spent less time in the hospital compared with *P. falciparum* patients (Table 1). Thrombocytopenia (platelet count <150,000/*µ*L) was identified as a predominant clinical parameter in patients with severe *P. vivax* and *P. falciparum* infections. Thrombocytopenia was observed in 55% of severe *P. vivax* cases and 68% of severe *P. falciparum* cases (Table 2). In both *P. vivax* and *P. falciparum* cases, approximately one-third of thrombocytopenia cases were severe (platelet count <50,000/*µ*L). The baseline platelet counts for the Indian population have been reported to be in the range of 138,000/*µ*L to 355,000/*µ*L.^33^ These observations suggest that the level of disease severity was greater for *P. falciparum* infections and that thrombocytopenia was a prominent clinical outcome for both severe *P. vivax* and *P. falciparum* cases.

### Discrepancy between parasitemia and parasite biomass levels indicates parasite accumulation, especially in severe *P. vivax* cases.

To assess the correlation between severe clinical phenotypes and parasitological factors, parasitemia and parasite biomass levels were measured in patients with *P. vivax* and *P. falciparum* infections. Peripheral parasitemia may have been affected by treatment; therefore, parasitemia levels were analyzed separately in untreated and treated *P. vivax* and *P. falciparum* patients. In untreated *P. vivax* patients, parasitemia levels were significantly higher in uncomplicated patients than in severe patients (Figure 1A). However, among untreated *P. falciparum* patients, parasitemia levels were significantly lower in uncomplicated versus severe patients (Figure 1B). Overall, across the dataset, uncomplicated *P. vivax* cases exhibited significantly higher parasitemia than severe *P. vivax* cases (Supplemental Figure 2A), whereas *P. falciparum* cases did not exhibit a significant difference between uncomplicated and severe cases (Supplemental Figure 2B; Table 1). Furthermore, parasitemia levels were only compared in samples from treated patients, in which parasitemia levels exhibited no significant difference between uncomplicated and severe cases in both *P. vivax* and *P. falciparum* infections (Supplemental Figure 2C and D).

**Figure 1.**
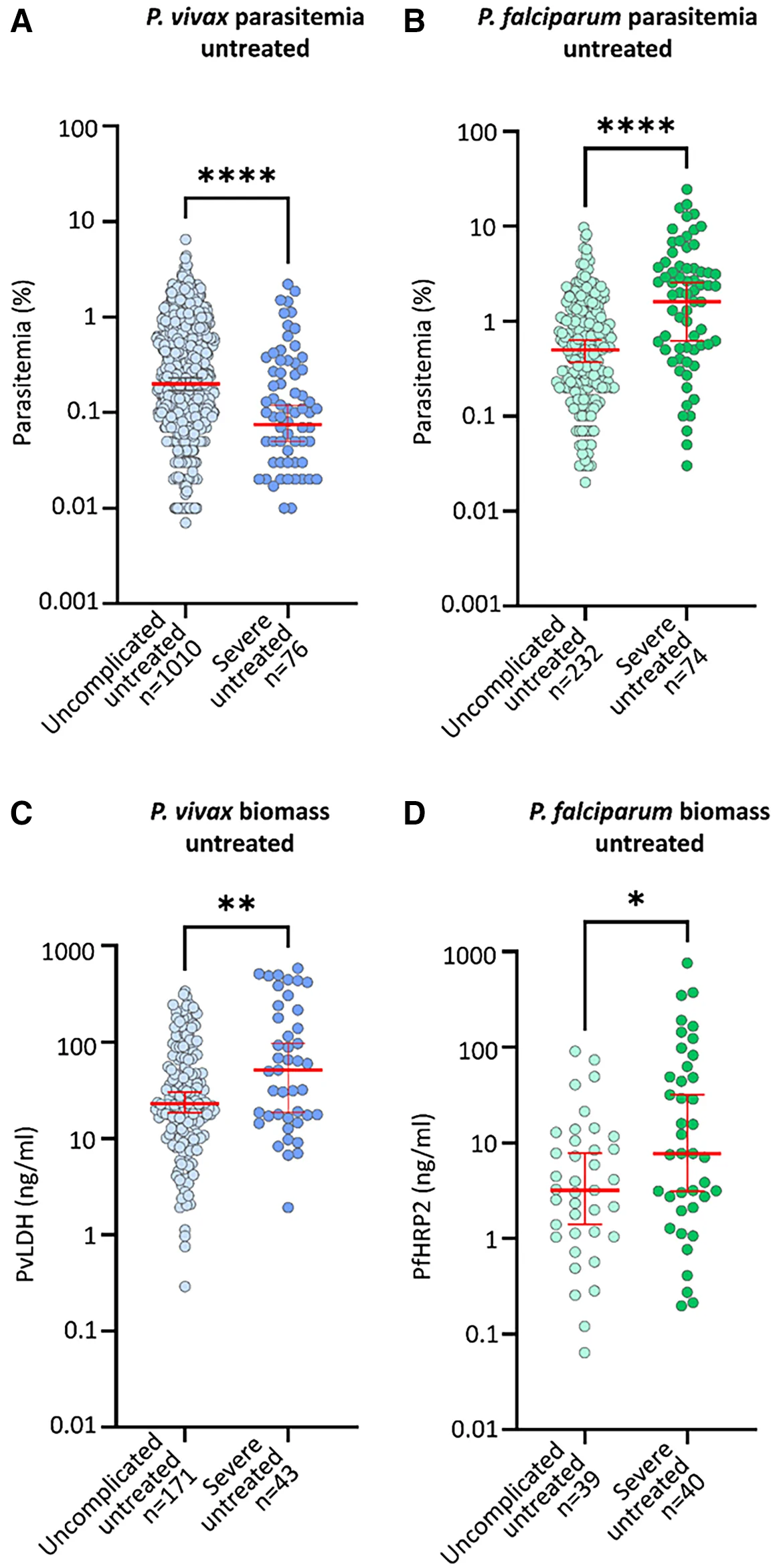
Comparison between uncomplicated and severe malaria patient samples in the untreated dataset showing (**A**) *Plasmodium vivax* (*P. vivax*) and (**B**) *Plasmodium falciparum* (*P. falciparum*) parasitemia levels, as well as (**C**) *P. vivax* lactate dehydrogenase levels in *P. vivax* and (**D**) PfHRP2 levels in *P. falciparum*. The median and its 95% CI are indicated by the red lines.

Next, levels of circulating antigens PvLDH for *P. vivax* and PfHRP2 for *P. falciparum* were measured as a proxy for total parasite biomass estimation. To achieve this, the authors randomly selected 22% and 24% of total *P. vivax* and *P. falciparum* patients, respectively. Parasite biomass measurements were unlikely to be affected by drug treatment because of the long half-lives of PfHRP2 (3.67 days) and PvLDH (22 hours) in patient blood.^12^^,^^34^ In the study dataset, for *P. vivax*, only 3% of the uncomplicated cases and 50% of the severe cases were treated, whereas for *P. falciparum*, ∼20% of the uncomplicated and 60% of the severe patients were treated before enrollment. Significantly higher parasite biomass was observed for both severe *P. vivax* (Figure 1C) and severe *P. falciparum* (Figure 1D) cases compared with respective uncomplicated cases, irrespective of whether samples from only untreated patients (Figure 1C and D) or all samples from treated and untreated patients (Supplemental Figure 2E and F; Table 1) were examined.

The combination of higher parasite biomass and lower parasitemia, particularly in severe *P. vivax* cases, suggests that peripheral parasitemia did not reflect parasite burden, either owing to pretreatment or to parasite accumulation in non-blood tissues. Further, a logistic multiple regression model assessing the odds of severe versus uncomplicated *vivax* malaria with respect to both log(parasitemia) and log(biomass) revealed that both variables had significant yet contrasting effects (*N* = 271; *P* <0.00001 for both). Specifically, a 2-fold increase in biomass increases the odds of severity 1.4-fold, whereas a 2-fold decrease in parasitemia also increases the odds of severity 1.4-fold, and both 2-fold changes together increase the odds of severity 2-fold. These observations indicate a discordance between peripheral parasitemia and total parasite biomass, underscoring the importance of hidden parasite biomass, especially for severe *P. vivax* cases. These observations imply that parasite biomass might be a more important factor in disease severity than parasitemia in severe malaria infections.

### Parasite biomass is robustly associated with the degree of malaria severity in severe *P. vivax* and *P. falciparum* cases.

Disease severity had been shown to be associated with parasite biomass and only very weakly associated with parasitemia in *P. falciparum* infections.^27^^,^^28^ When the association between parasitemia/parasite biomass and disease severity in severe *P. vivax* and *P. falciparum* patients was investigated, the degree of severity (severity criteria, treated as a continuous variable) was observed to be negatively correlated with parasitemia in *P. vivax* cases (Figure 2A; Spearman *r* = −0.11, *P* = 0.1269), whereas for *P. falciparum* cases, it was positively correlated (Figure 2B; Spearman *r* = 0.16, *P* = 0.0199). Furthermore, the degree of severity was positively correlated with parasite biomass in *P. vivax* cases (Figure 2C; Spearman *r* = 0.26, *P* = 0.0088) as well as in *P. falciparum* cases (Figure 2D; Spearman *r* = 0.49, *P* <0.0001). This pattern suggests the accumulation of parasites in *P. vivax* cases, which accounts for the higher parasite biomass. A similar pattern was observed for the association between degree of severity and parasitemia when only a subset of cases for which parasite biomass values were known was considered (Supplemental Figure 3A and B). This pattern did not change when untreated patients were analyzed in a similar manner to rule out the effect of pretreatment (Supplemental Figure 3C and D). These observations are in agreement with previous reports and clearly indicate that parasite biomass, rather than parasitemia, is a robust parasitological parameter associated with severity in *P. vivax* and *P. falciparum* malaria.^10^^,^^13^^,^^23^^,^^25^

**Figure 2.**
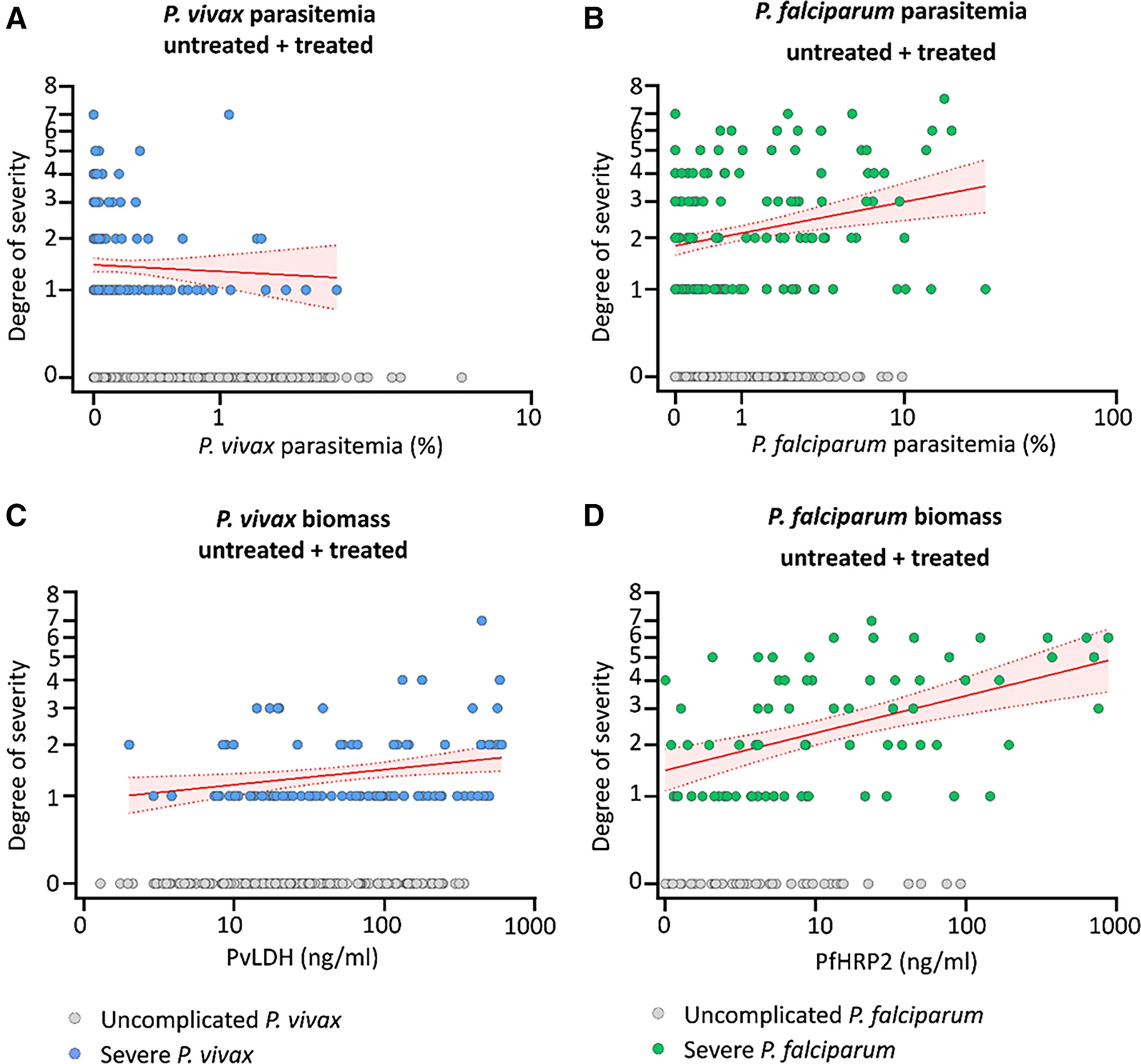
Association of the degree of severity (severity criteria) with parasitemia and parasite biomass in the untreated and treated dataset (severe cases only). Association analysis of parasitemia with severity revealed (**A**) a weakly negative linear trend (*n* = 170, slope = −0.07, *R*^2^ = 0.003, *P* = 0.47; Spearman *r* = −0.11, *P* = 0.12) in *Plasmodium vivax* (*P. vivax*)-infected patients, and (**B**) a positive regression correlation (*n* = 197, slope = 0.14, *R*^2^ = 0.06, *P* = 0.0002) and a positive nonparametric correlation (Spearman *r* = 0.16, *P* = 0.01) in *Plasmodium falciparum*-infected patients. Association analysis of parasite biomass with degree of severity revealed (**C**) a positive regression correlation (*n* = 100, slope = 0.05, *R*^2^ = 0.06, *P* = 0.0089) and a positive nonparametric correlation (Spearman *r* = 0.26, *P* = 0.0088) in *P. vivax*, as well as (**D**) a positive regression correlation (*n* = 74, slope = 0.12, *R*^2^ = 0.23, *P* <0.0001) and a positive nonparametric correlation (Spearman *r* = 0.49, *P* <0.0001). Relevant levels for uncomplicated patients in each panel that were not included in the association analyses are represented by gray circles to show the overall distribution. The red solid line represents the best-fit line determined by linear regression and indicates the relationship between two variables (i.e., degree of severity with *P. vivax* lactate dehydrogenase/PfHRP2 or parasitemia). The degree of severity is represented on a scale of 1 to 8, with 1 being the least severe and 8 being the most severe. The red shaded area represents the 95% CI for the best-fit line.

### Parasite biomass is associated with thrombocytopenia and severity in *P. vivax and P. falciparum* malaria cases.

Thrombocytopenia (lower platelet counts) has been reported frequently with severe malaria^15^^,^^35^^,^^36^ and has been associated with higher parasite biomass, improving the severe malaria diagnosis in *P. falciparum* patients in Africa.^37^ In the study dataset, thrombocytopenia was also identified as a highly prevalent clinical feature for both *P. vivax* and *P. falciparum* malaria patients (Table 2). This led the authors to investigate whether parasite biomass, thrombocytopenia, and disease severity are interconnected. They found that the platelet counts were inversely correlated with parasite biomass in both *P. vivax* (Figure 3A; Spearman *r* = −0.22, *P* = 0.0345) and *P. falciparum* cases (Figure 3B; Spearman *r* = −0.33, *P* = 0.0041). For severe *P. vivax* cases, platelet counts were inversely associated with the degree of severity, although the correlation was not statistically significant (Figure 3C; Spearman *r* = −0.14, *P* = 0.1042). For severe *P. falciparum* cases, however, platelet counts were inversely associated with the degree of severity (Figure 3D; Spearman *r* = −0.30, *P* <0.0001). These observations indicated that higher parasite biomass was associated with thrombocytopenia in both severe *P. vivax* and *P. falciparum* patients, whereas thrombocytopenia was associated with the degree of severity only in severe *P. falciparum* patients. Although not statistically significant, thrombocytopenia in severe *P. vivax* cases exhibited a positive trend with the degree of severity.

**Figure 3.**
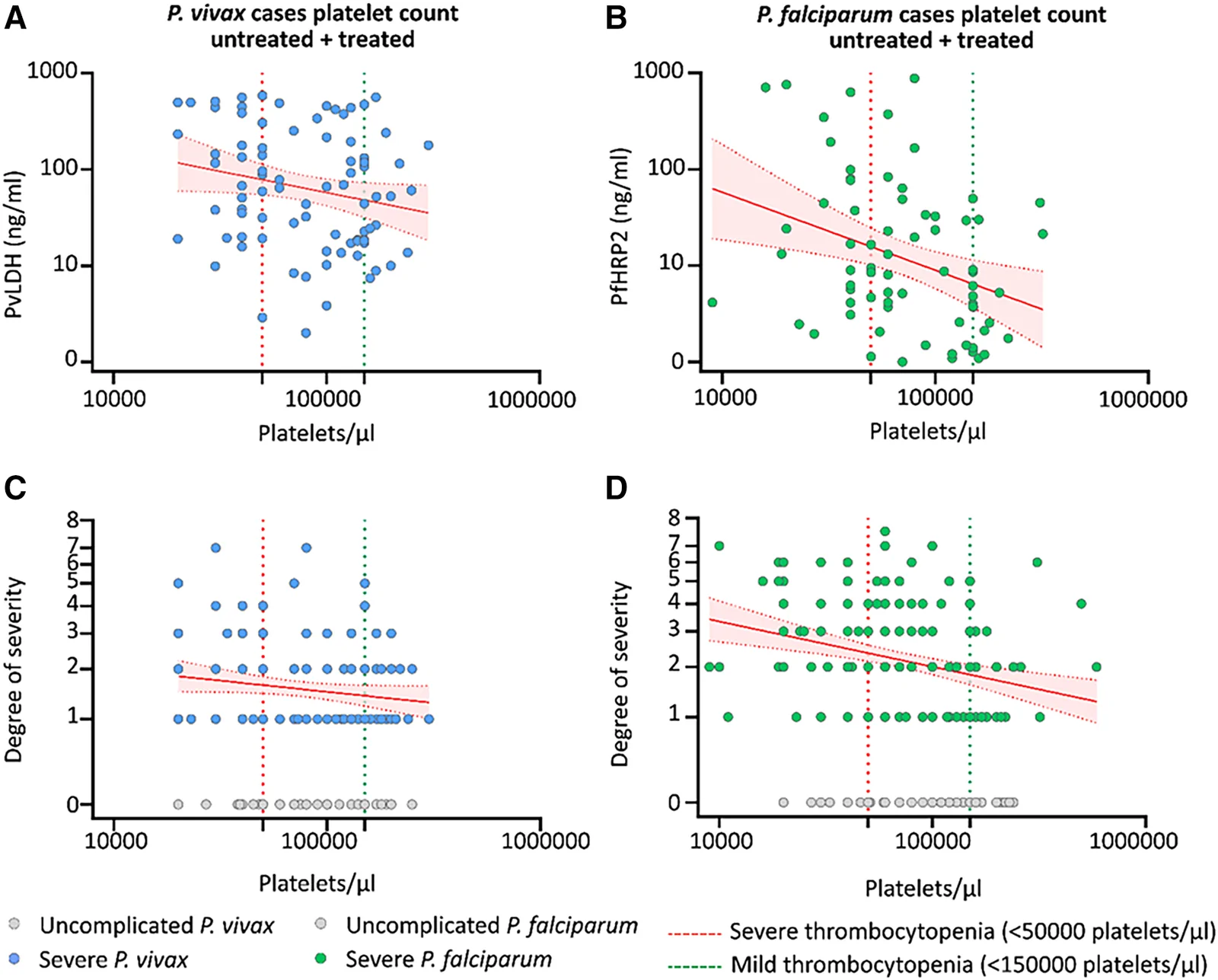
Association of platelet counts with parasite biomass and degree of severity in the untreated and treated dataset. Linear regression and correlation analyses revealed (**A**) a negative regression correlation (*n* = 86, slope = −0.44, *R*^2^ = 0.04, *P* = 0.05) and negative nonparametric correlation (Spearman *r* = −0.22, *P* = 0.03) between platelet count and *Plasmodium vivax* (*P. vivax*) lactate dehydrogenase levels in *P. vivax*, (**B**) a negative regression correlation (*n* = 72, slope = −0.80, *R*^2^ = 0.11, *P* = 0.004) and negative nonparametric correlation (Spearman *r* = −0.33, *P* = 0.004) between platelet count and PfHRP2 levels in *Plasmodium falciparum* (*P. falciparum*), (**C**) a weakly negative linear trend (*n* = 130, slope = −0.07, *R*^2^ = 0.02, *P* = 0.07; Spearman *r* = −0.14, *P* = 0.10) of platelet count with degree of severity in *P. vivax*-infected patients and (**D**) a negative regression correlation (*n* = 179, slope = −0.15, *R*^2^ = 0.08, *P* <0.0001) and negative nonparametric correlation (Spearman *r* = −0.30, *P* <0.0001) of platelet count with degree of severity in *P. falciparum*-infected patients. Relevant levels for uncomplicated patients in each panel that were not included in the association analyses are represented by gray circles to show the overall distribution. The red solid line represents the best-fit line determined by linear regression. The degree of severity is represented on a scale of 1 to 8, with 1 being the least severe and 8 being the most severe. The red shaded area represents the 95% CI for the best-fit line.

### Severe malaria patients with thrombocytopenia and higher parasite biomass spend more time in the hospital.

The length of stay of malaria patients at health centers is an important factor for effective case management, and certain clinical risk factors were shown to be associated with it.^38^^,^^39^ Although important in malaria case management, factors affecting the length of hospital stay of malaria patients are not well understood. A longer hospital stay was strongly associated with disease severity in both *P. vivax* and *P. falciparum* cases (Supplemental Table 1). A significant association was also observed between parasite biomass and length of hospital stay for both severe *P. vivax* (Figure 4A; Spearman *r* = 0.28, *P* <0.0001) and *P. falciparum* cases (Figure 4B; Spearman *r* = 0.30, *P* = 0.0011). The average time spent in the hospital was slightly higher in severe *P. falciparum* patients compared with *P. vivax* patients (Table 1), indicating that severe *P. falciparum* patients with higher parasite biomass took longer to recover. Significant negative associations were observed between length of hospital stay and parasitemia for both parasite species (Supplemental Figure 4). Furthermore, platelet counts were negatively correlated with the duration of hospital stay in *P. vivax* (Figure 4C; Spearman *r* = −0.1682, *P* = 0.0221) and *P. falciparum* cases (Figure 4D; Spearman *r* = −0.2661, *P* <0.0001). Although the utility of thrombocytopenia alone as a prognostic marker for malaria cases is limited,^35^ the present study indicates that the use of thrombocytopenia together with parasite biomass might be effective for the prognosis, triage, and management of severe malaria.

**Figure 4.**
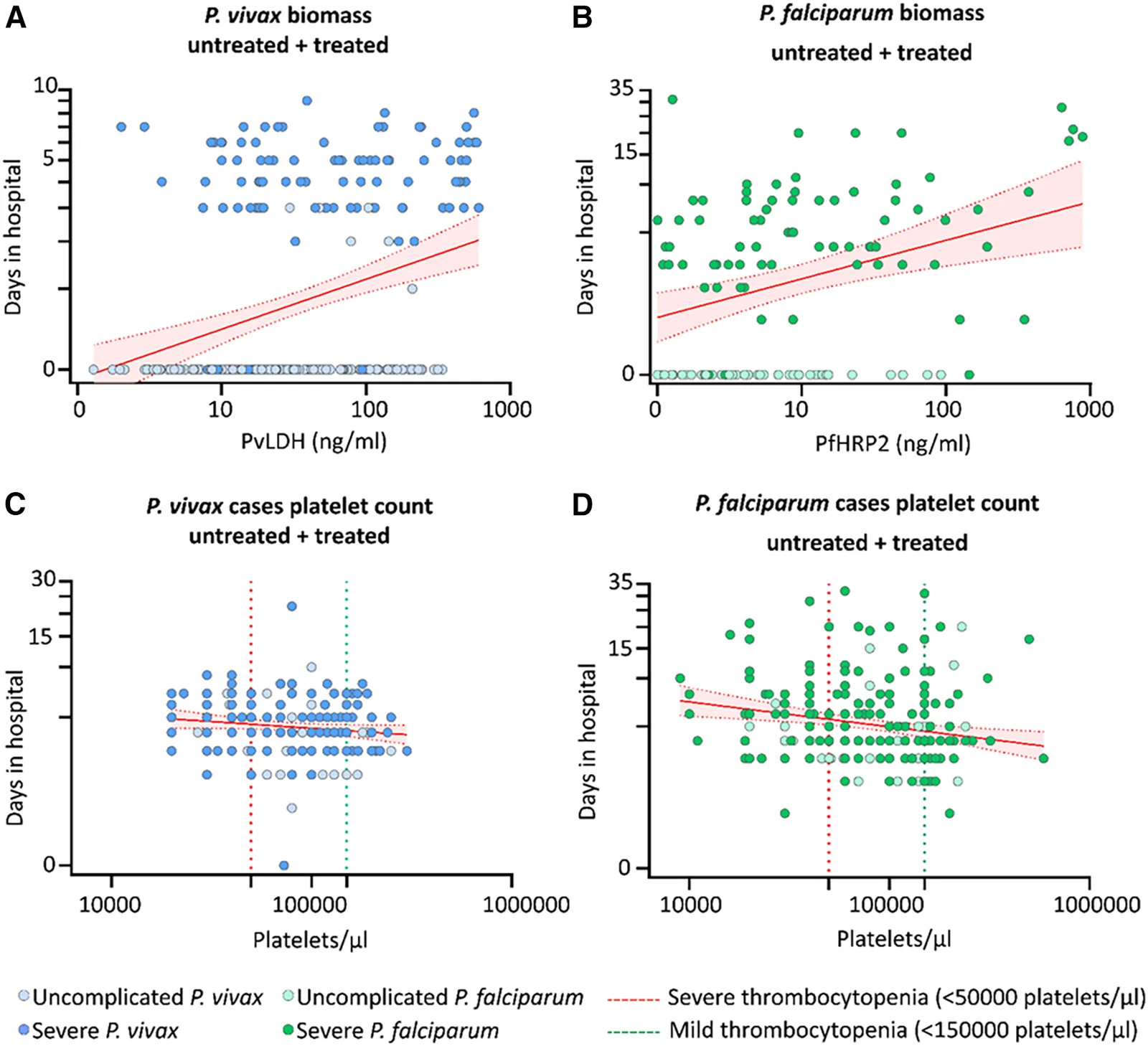
Association between length of hospital stay and parasite biomass and platelet counts in the untreated and treated dataset. Linear regression and correlation analyses revealed (**A**) a positive regression correlation (*n* = 273, slope = 0.18, *R*^2^ = 0.09, *P* <0.0001) and positive nonparametric correlation (Spearman *r* = 0.28, *P* <0.0001) between *Plasmodium vivax* (*P. vivax*) lactate dehydrogenase (PvLDH) levels and days in hospital in *P. vivax*, (**B**) a positive regression correlation (*n* = 113, slope = 0.21, *R*^2^ = 0.11, *P* = 0.0003) and a positive nonparametric correlation (Spearman *r* = 0.30, *P* = 0.001) between PfHRP2 levels and days in the hospital in *Plasmodium falciparum* (*P. falciparum*), (**C**) a weak negative linear trend (*n* = 185, slope = −0.000004356, *R*^2^ = 0.1395, *P* = 0.1093) and a negative nonparametric correlation (Spearman *r* = −0.16, *P* = 0.02) between platelet counts and days in the hospital in *P. vivax*, and (**D**) a negative regression correlation (*n* = 243, slope = −0.13, *R*^2^ = 0.04, *P* = 0.001) and negative nonparametric correlation (Spearman *r* = −0.26, *P* <0.0001) between platelet counts and days in the hospital in *P. falciparum* infected patients. The red solid line represents the best-fit line determined by linear regression between days in the hospital and PvLDH/PfHRP2 or platelets. The red shaded area represents the 95% CI for the best-fit line.

### Multivariate analysis indicates key clinical parameters important for severity and length of hospital stay.

To identify key clinical parameters that might be important for severity and length of hospital stay, LASSO models were used to test various clinical parameters for association with disease outcomes (Table 3). The models were constructed using two different datasets, both restricted to inpatients: the subset containing patient samples for which parasite biomass data are available versus the whole set of inpatient samples (Table 1). In contrast to the univariate analysis, for LASSO models, only patient samples with comprehensive clinical data were considered. For *P. vivax* severity, there were no significant multivariate models; the abovementioned significant multivariate model incorporating parasitemia and biomass (*n* = 271) was not significant in this reduced set of inpatient samples with all clinical parameters used for LASSO analysis (*n* = 89). For days spent in the hospital among *P. vivax* patients, parasitemia, platelets, radiologically confirmed pulmonary edema, systolic BP, respiration rate, total bilirubin levels, and serum creatinine levels were the most important parameters. For *P. falciparum* severity, parasite biomass, parasitemia, and platelet count were all top parameters. For days spent in the hospital among *P. falciparum* patients, parasite biomass, serum creatinine levels, total bilirubin levels, systolic BP, and HCT levels all contributed to significant models. Although parasite biomass and thrombocytopenia individually exhibit associations with severity and time spent at hospital, the only significant multivariate model containing both of these variables was found for *P. falciparum* severity, and only if severity was modeled as binary: odds of severity = exp (10.711 + 0.336 × log [PfHRP2 + 0.01] − 0.9194 × log [platelets] − 0.423 × pretreatment; *P* <0.05 for PfHRP2 and platelets). Taken together, these multivariate results indicate that complex predictive models with several clinical parameters can outperform univariate models, but only for certain disease outcomes. A larger number of patient samples could reveal additional models for which the limited dataset is insufficient.

**Table 3:** Clinical factors correlated with disease outcomes (severity and days patients spent in the hospital) for *Plasmodium vivax* and *Plasmodium falciparum*

Species/Outcome	Dataset (*n*)	Single Parameter (parasitemia/ parasite biomass)	Best AIC	Second-Best Multivariate AIC	Third-Best Multivariate AIC
*P. vivax* severity					
	Inpatient data with PvLDH (89)	Biomass	–	–	–
Best multivariate model: None					
	All inpatient data (134)	–	–	–	–
Best multivariate model: None					
*P. vivax* days spent in the hospital					
	Inpatient data with PvLDH (95)	Parasitemia	Parasitemia + pulmonary edema + pretreatment	Parasitemia + platelets + pretreatment	–
Best multivariate model: Days = exp (1.554 + 0.061 × log (parasitemia + 0.01) + 0.443 × (pulmonary oedema) + 0.099 × pretreatment)					
	All inpatient data (190)	–	Pulmonary edema + systolic BP + hemoglobin + serum creatinine + pretreatment	Pulmonary edema + hemoglobin + serum creatinine + pretreatment	Pulmonary edema + systolic BP + hemoglobin + pretreatment
Best multivariate model: Days = exp (2.351 + 0.373 × (pulmonary edema) − 0.004 × (systolic BP) − 0.049 × hemoglobin + 0.144 × (serum creatinine) − 0.003 × pretreatment)					
*P. falciparum* severity					
	Inpatient data with PfHRP2 (71)	Biomass	Parasitemia + PfHRP2 + pretreatment	Parasitemia + platelets + pretreatment	–
Best multivariate model: Severity = exp (0.476 + 0.136 × log (parasitemia + 0.01) + 0.117 × log (PfHRP2 + 0.01) + 0.405 × pretreatment)					
	All inpatient data (183)	–	Parasitemia + platelets + pretreatment	Platelets + age + pretreatment	Parasitemia + age + pretreatment
Best multivariate model: Severity = exp (2.941 + 0.061 × log (parasitemia + 0.01) − 0.200 × log (platelets) + 0.165 × pretreatment)					
*P. falciparum* days spent in hospital					
	Inpatient data with PfHRP2 (71)	Biomass	Serum creatinine + total bilirubin + systolic BP + pretreatment	Serum creatinine + systolic BP + pretreatment	Total bilirubin + systolic BP + pretreatment
Best multivariate model: Days = exp (3.093 + 0.264 × log (serum creatinine) + 0.147 × log (total bilirubin) − 0.019 × (systolic BP) + 0.087 × pretreatment)					
	All inpatient data (247)	–	Serum creatinine + total bilirubin + systolic BP + pretreatment	Serum creatinine + total bilirubin + pulmonary edema + pretreatment	Serum creatinine + hematocrit + systolic BP + pretreatment
Best multivariate model: Days = exp (2.072 + 0.292 × log (serum creatinine) + 0.096 × log (total bilirubin) − 0.007 × (systolic BP) + 0.132 × pretreatment)					

AIC = akaike information criterion; BP = blood pressure; exp = the exponential function e^x^; *n* = number of patients included in the analysis; *P. falciparum* = *Plasmodium falciparum*; *P. vivax* = *Plasmodium vivax*; PfHRP2 = *P. falciparum* histidine-rich protein 2; PvLDH = *P. vivax* lactate dehydrogenase.

The authors ran models either by excluding patient samples with missing biomass data (“Inpatient data with PvLDH” or “Inpatient data with PfHRP2”) or by including all patient samples but excluding biomass as a potential parameter (“All inpatient data”). For the former scenario, they tested whether biomass or parasitemia alone, with only pretreatment as the other independent variable, yielded a lower AIC (“Single parameter”). For all scenarios, they fit multivariate LASSO models, forcing pretreatment as a binary parameter in all models, regardless of whether it had a significant effect. They show the model with the lowest AIC and with all its parameters being significant (other than pretreatment), if such a model could be identified (“Best AIC”). If the best model has at least two significant parameters other than pretreatment, the complete parameters of the model with the lowest AIC are shown (“Best multivariate model”), and up to two additional models that meet these criteria are listed if they exist.

## DISCUSSION

Historically, Indian *P. falciparum* infections have been reported to be more severe than *P. vivax* infections.^24^ However, severe *P. vivax* infections are now well recognized as a serious problem in India.^3^^,^^36^^,^^40^^,^^41^ In a low-transmission setting like India, malaria severity in *P. falciparum* is observed primarily in adults, similar to that observed in Southeast Asia, but in contrast to the predominantly pediatric malaria observed in sub-Saharan Africa.^42^ Jaundice, renal impairment, and shock were the primary clinical outcomes seen in severe *P. falciparum* patients in the present study, similar to those observed in Thailand.^43^ In the present study, 21% of enrolled study participants presented with severe malaria. Of these severe malaria patients, 13.4% and 69.4% had *P. vivax* and *P. falciparum* infections, respectively, whereas the remainder had coinfections. Severe *P. vivax* malaria patients presenting with anemia, renal impairment, radiologically confirmed pulmonary edema, and thrombocytopenia were similar to those observed in severe *P. falciparum* malaria, although the severity of these features was greater in *P. falciparum* malaria.

In the case of *P. falciparum*, substantial evidence has been accrued indicating a strong association between severity and higher parasite biomass, with parasite biomass providing a better predictive value than parasitemia for severity.^27^^,^^44^ The present study reinforces this observation for *P. falciparum*. The average prevalence of PfHRP2 gene deletion in Indian samples from central India has been reported to be ∼4%, and the data for PfHRP2 gene deletions are not available specifically for the Goan population.^45^^,^^46^ In contrast, studies in which the role of parasite biomass has been investigated in *P. vivax* pathogenesis have been limited.^11^^,^^47^ With a relatively large dataset in the present study, the authors conclusively demonstrate that severe *P. vivax* patients harbor higher parasite biomass compared with their uncomplicated counterparts, suggesting a strong role for parasite accumulation in the pathology of severe *P. vivax* malaria. A recent report has revealed high levels of parasite accumulation in low-density *P. vivax* infections.^10^ The present study’s data suggest this to be true for higher density clinical infections, as well as indicating *P. vivax* accumulation in specific organs such as the spleen, bone marrow, and lungs.^10^^,^^14^^,^^21^^,^^48^^,^^49^ These organs with hidden parasite reservoirs might be sites of disturbances in hematologic homeostasis that cause inflammation and endothelial cell (EC) activation.^14^^,^^48^^,^^50^ In fact, elevated levels of pro-inflammatory cytokines such as tumor necrosis factor, interferon-γ, interleukin (IL)-6, IL-8, IL-18, and monocyte chemo-attractant protein-1,^15^^,^^51^ as well as the autocrine mediator of EC activation, angiopoietin-2, have been found to be elevated in severe *P. vivax* infections.^52^ Furthermore, the authors’ observations indicate that parasite biomass measured using PvLDH/PfHRP2 as a predictor of severe malaria has utility over parasitemia, which is the conventional diagnostic parasitological factor. Additionally, parasite biomass (measured using PvLDH/PfHRP2 markers) could provide further insights into assessing severity in *P. vivax* and *P. falciparum* infections.

Alongside parasite biomass, thrombocytopenia has been shown to be an important diagnostic marker for severe *P. falciparum* malaria in Africa.^37^ Although it is not included in the WHO criteria for severity classification, thrombocytopenia has been shown to be associated with malaria severity in both *P. vivax* and *P. falciparum* infections.^17^^,^^18^ A positive correlation between thrombocytopenia and disease severity for *P. falciparum* was also found in the present study, suggesting its role in disease prognosis. A similar (although nonsignificant) trend was also observed in *P. vivax* cases, as reported previously.^18^ Platelets play an important role in the immune response to malaria infection because they have been shown to kill parasites through splenic clearance, heightening levels of inflammatory cytokines and endothelial activation.^39^^,^^40^ Thrombocytopenia is common in individuals infected with both *P. vivax* and *P. falciparum.*^21^^,^^53^^–^^56^ The study findings suggest that thrombocytopenia can be a strong predictor, comparable to parasite biomass, particularly in severe *P. falciparum* cases. Thus, the quantification of parasite biomass^57^^,^^58^ as a clinical point-of-care parameter, along with thrombocytopenia, might serve as an important indicator for *P. falciparum* malaria severity.

The present study indicates that parasitemia was negatively associated with hospital stay in both *P. vivax* and *P. falciparum* patients, suggesting that parasite biomass is a more reliable indicator of severity and longer hospital stay. However, more studies with a greater number of severe *P. vivax* patients are needed to firmly establish this link. The authors further considered combinatorial predictors of disease severity and hospital stay and found limited improvement in predictive value beyond the univariate models. The LASSO model indicated that for *P. vivax*, radiologically confirmed pulmonary edema, systolic BP, respiration rate, platelets, total bilirubin levels, and serum creatinine levels were the most important parameters predicting hospital stay. High mortality has been observed in severe *P. vivax* patients with radiologically confirmed pulmonary edema and increased respiration rate.^48^^,^^59^ Importantly, positive associations between higher parasite biomass and thrombocytopenia and longer hospital stay have been demonstrated for *P. vivax*, further underscoring the importance of these factors in clinical outcomes.

## CONCLUSION

Although patients with comorbidities were excluded at enrollment, concurrent acute pathologies were not systematically assessed in the present study; therefore, severe symptoms arising from other acute pathologies cannot be completely ruled out. Furthermore, the authors’ inability to measure levels of EC activation biomarkers and drug levels (in pretreated cases) in study participants was another limitation. Although they used a subset of a larger dataset for parasite biomass estimation, effect size analysis indicates that this dataset had the power to detect a 1.6-fold difference in PvLDH between severe and uncomplicated cases, whereas the actual difference we detected was 2.2-fold. Similarly, for *P. falciparum*, the dataset had the power to detect a 2.4-fold difference in PfHRP2 between severe and uncomplicated cases, whereas the actual difference we detected was 3.4-fold. Even with these limitations, a higher parasite load, particularly in severe *P. vivax* malaria cases, was identified with a large dataset for the first time in India. Additionally, the study data suggest that the combination of higher parasite biomass with thrombocytopenia might be important for predicting severe clinical outcomes of *P. vivax* and *P. falciparum* infection. Further large-scale studies are needed to establish the combined association between parasite biomass and thrombocytopenia and severity, particularly for *P. vivax* malaria. Nonetheless, the present study highlights the association between high parasite biomass and thrombocytopenia and severity and increased length of hospitalization for *P. vivax* and *P. falciparum* malaria, which might prove effective for the prognosis, triage, and management of severe malaria.

## Supplemental Materials

10.4269/ajtmh.26-0106 Supplemental Materials
