# Supplementary material for: Association of Parasite Biomass with Disease Severity in Plasmodium vivax and Plasmodium falciparum Malaria Infections
Source: Am J Trop Med Hyg. 2026 Aug 27;115(4):795–806. doi: 10.4269/ajtmh.26-0106 (PMC13527199; doi:10.4269/ajtmh.26-0106)
Supplement: Supplementary file 1 [file tpmd260106.SD1.pdf]

Supplementary Figure 1: CONSORT diagram outlining the study workflow including screening, enrolment and analysis.

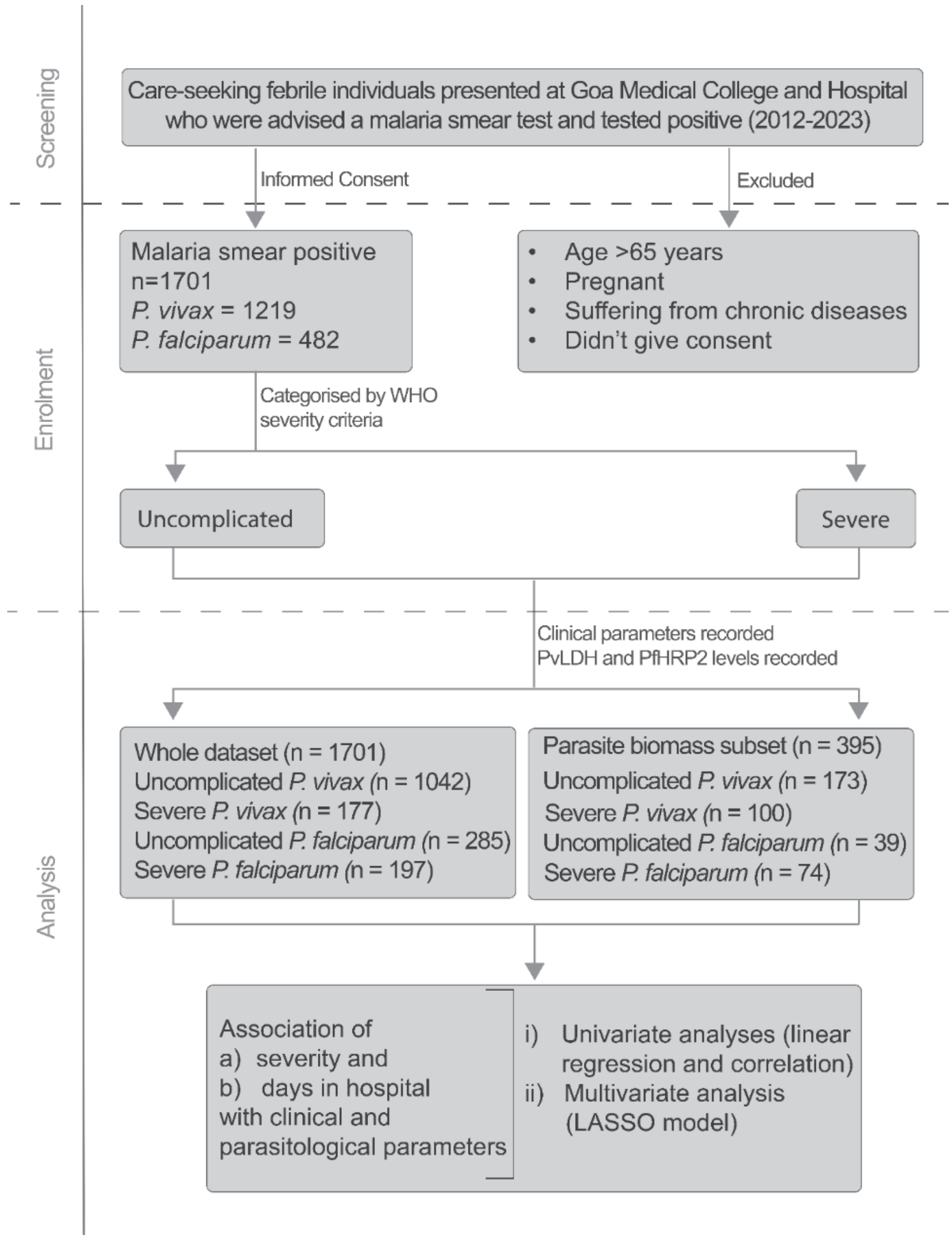

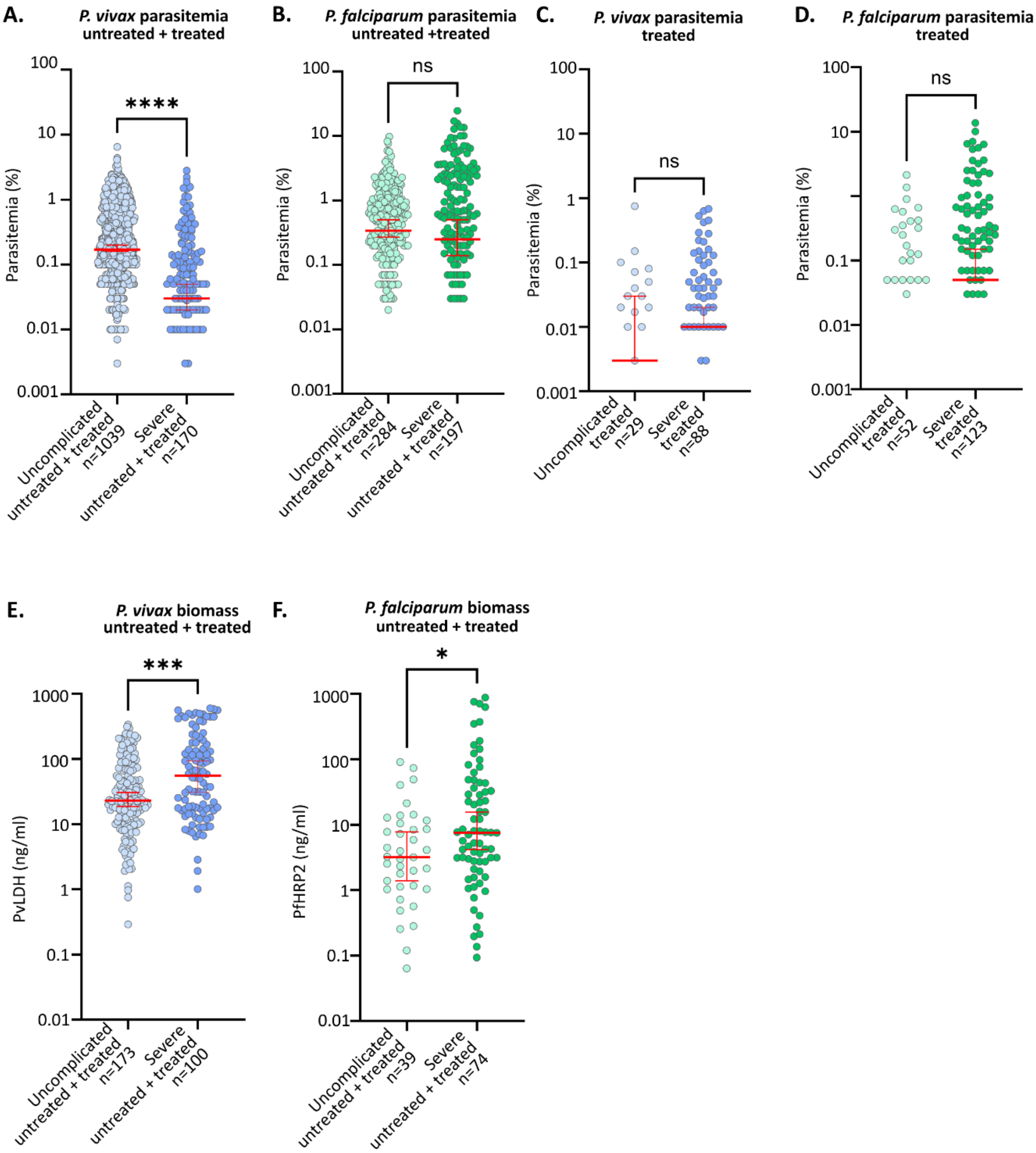

Supplementary Figure 2: Comparison of parasitemia and parasite biomass levels between uncomplicated and severe samples. Parasitemia levels in untreated + treated (A) *P. vivax* and, (B) *P. falciparum* patients compared to treated (C) *P. vivax* and, (D) *P. falciparum* patients point to Simpson's paradox. Parasite biomass in (E) untreated + treated *P. vivax* patients is depicted through PvLDH levels and, (F) in untreated + treated *P. falciparum* patients through PfHRP2 levels in. The positive association with severity in untreated *P. falciparum* (Fig 2D) is obscured in this larger dataset,

suggesting treatment to be a possible confounding variable. Bivariate comparison was done using non-parametric Mann-Whitney U test. Median with 95% confidence interval is indicated by the red lines. Median for uncomplicated + treated samples at 0 in panel F is not shown.

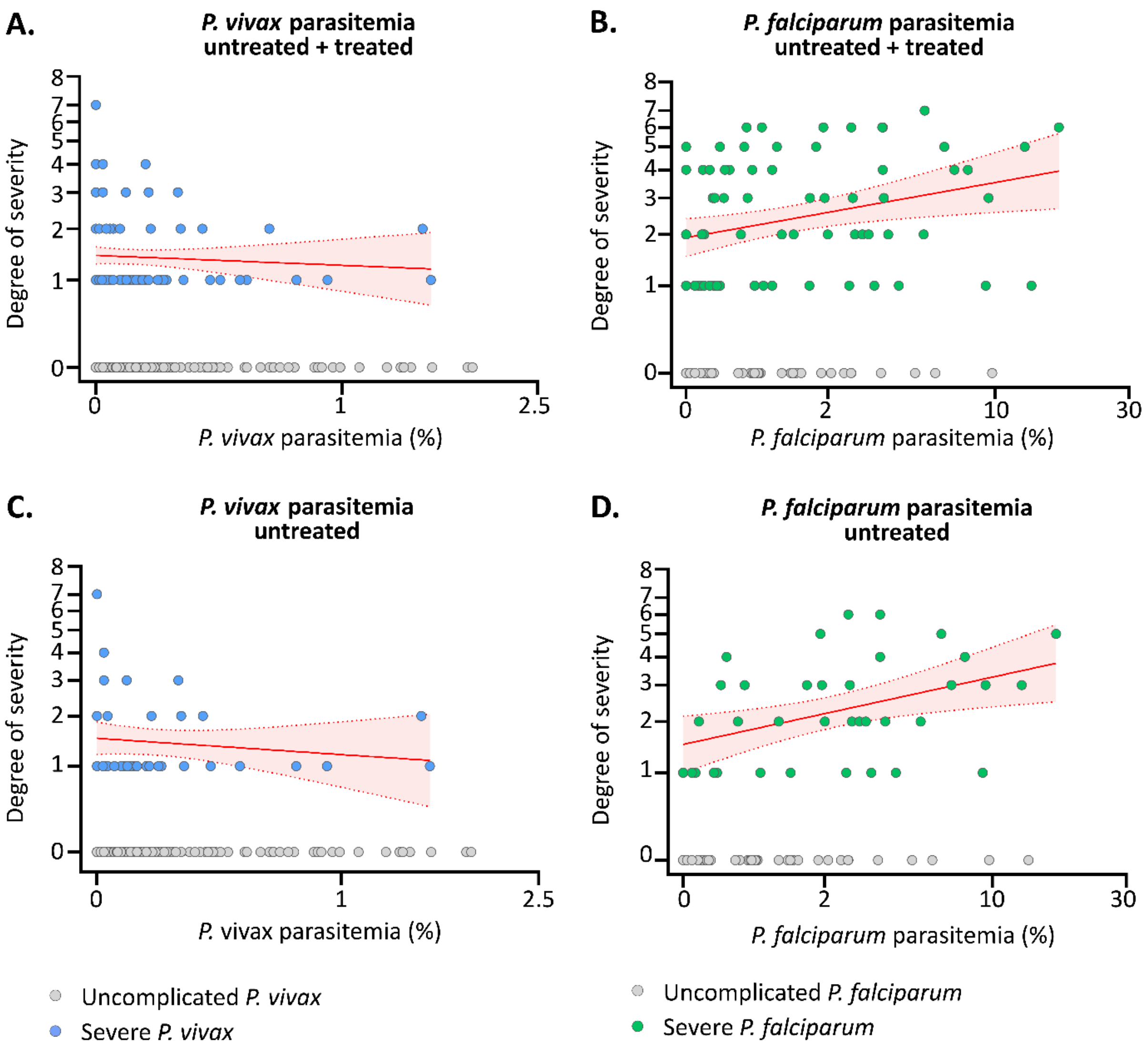

Supplementary Figure 3: Association of parasitemia with degree of severity for a subset for which PvLDH/PfHRP2 values were determined in untreated + treated, and untreated dataset, only severe samples. Linear regression and correlation analyses in untreated+ treated dataset revealed (A) a weak negative linear trend (n=98, slope= -0.11, R squared= 0.004, p= 0.50; Spearman r= -0.11, p= 0.26) between *P. vivax* parasitemia and degree of severity, and (B) a positive regression correlation (n= 74, slope= 0.18, R squared= 0.09, p= 0.008) and a positive nonparametric correlation (Spearman r= 0.34, p value= 0.0027) between *P. falciparum* parasitemia and degree of severity. Linear regression and correlation analysis in untreated dataset showed (C)

a weak negative trend (n=41,slope = -0.18, R squared= 0.01, p= 0.41; Spearman r= -0.22, p= 0.16) between *P. vivax* parasitemia and degree of severity (D) a positive regression correlation (n= 37, slope= 0.22, R squared= 0.18, p= 0.008) and a positive correlation (Spearman r= 0.42, p = 0.008) between *P. falciparum* parasitemia and degree of severity. Uncomplicated samples in each panel, which were not included in the association analyses, are represented by grey circles, to show overall distribution of samples. The red solid line represents the best-fit line determined by linear regression of parasitemia vs degree of severity. The degree of severity is represented on a scale of 0 to 8, where 0 denotes uncomplicated malaria, while the severity values 1 to 8 represent severity, 1 being least to 8 being most severe. The red shaded area denotes the 95% confidence interval for the best-fit line.

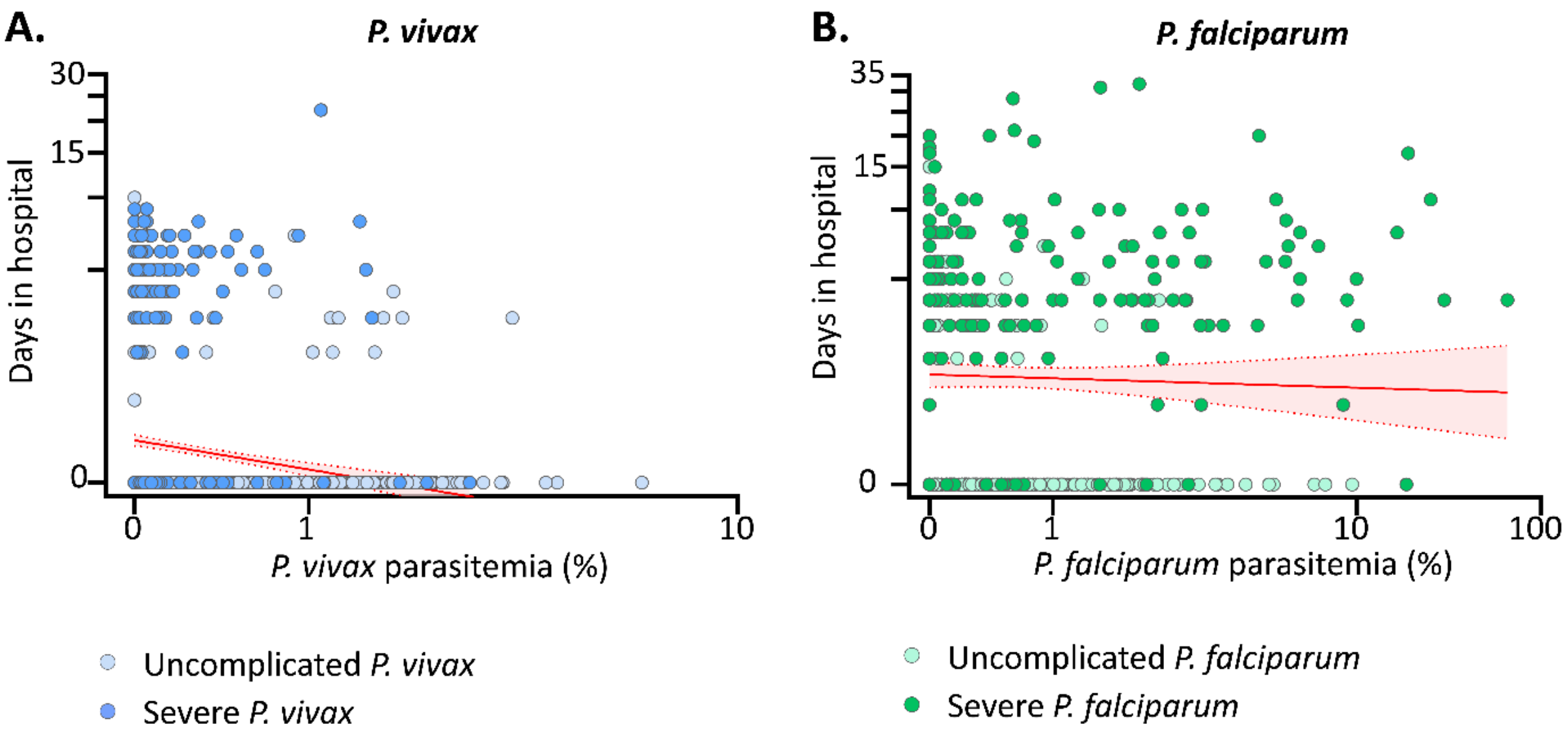

Supplementary Figure 4: Association of length of hospital stay with parasitemia in untreated+ treated dataset. Linear regression and correlation analyses revealed (a) negative regression correlation (n= 1209, slope= -0.35, R squared= 0.03, p= <0.0001) and negative nonparametric correlation (Spearman r= -0.28, p value= <0.0001) between *P. vivax* parasitemia and days in hospital, and (B) negative linear trend (n= 480, slope= -0.04, R squared= 0.0008, p=0.51) and negative nonparametric correlation (Spearman r= -0.22, p<0.0001) of length of hospital stay with *P. falciparum* parasitemia. The red solid line represents the best-fit line determined by linear regression. The red shaded area denotes the 95% confidence interval for the best-fit line.

Parameters	*P. vivax* severity			*P. vivax* days in hospital			*P. falciparum* severity			*P. falciparum* days in hospital		
	Spearman r	p value	n	Spearman r	p value	n	Spearman r	p value	n	Spearman r	p value	n
**Parasitemia**	-0.1175	0.1269	170	-0.2821	<0.0001	1207	0.1658	0.0199	197	-0.2224	<0.0001	480
**Parasitemia***	-0.1126	0.2696	98	0.0732	0.2297	271	0.3434	0.0027	74	0.0430	0.6512	113
**Parasite density**	-0.0720	0.3493	171	-0.1907	<0.0001	1208	0.1174	0.1005	197	-0.3232	<0.0001	478
**Parasite density***	-0.0758	0.4560	99	-0.3297	<0.0001	272	0.1906	0.1038	74	-0.0800	0.3997	113
**Gametocytemia**	-0.0349	0.6508	171	-0.0474	0.1000	1208	0.0787	0.2718	197	-0.0365	0.4266	478
**Gametocytemia***	-0.0228	0.8225	99	-0.0246	0.6868	272	0.1939	0.0978	0.098	-0.0529	0.5781	113
**Gametocyte density**	-0.0160	0.8356	171	-0.0857	0.0029	1209	0.0525	0.4639	197	-0.0303	0.5093	478
**Gametocyte density***	0.0578	0.5697	99	-0.0646	0.2882	272	0.0268	0.8208	74	-0.0871	0.3588	113
**Parasite biomass**	0.2607	0.0088	100	0.2826	<0.0001	273	0.4923	<0.0001	74	0.3023	0.0011	113
**Haemoglobin at enrolment**	0.1021	0.1854	170	-0.2185	<0.0001	1190	-0.2673	0.0003	177	-0.3051	<0.0001	440
**Hamoglobin at enrolment***	0.0780	0.4430	99	-0.2973	<0.0001	272	-0.5293	<0.0001	61	-0.4273	<0.0001	97
**Haematocrit at enrolment**	0.1075	0.1706	164	-0.1768	<0.0001	1148	-0.1858	0.0176	163	-0.2583	<0.0001	393
**Haematocrit at enrolment***	-0.0165	0.8723	97	-0.2051	0.0007	268	-0.3412	0.0133	52	-0.4385	<0.0001	85
**Platelets/ microlit**	-0.1431	0.1042	130	-0.1682	0.0221	185	-0.3377	<0.0001	173	-0.2661	<0.0001	243
**Platelets/ microlit ***	-0.1660	0.1290	85	-0.1841	0.0807	91	-0.3269	0.0065	68	-0.2738	0.0239	68
**Systolic BP**	-0.2013	0.0202	133	-0.1879	0.0096	189	0.0047	0.9493	183	-0.0919	0.1500	247
**Systolic BP***	-0.1698	0.1137	88	-0.1750	0.0933	93	-0.1206	0.3166	71	-0.2626	0.0269	71
**Blood Glucose**	-0.2796	0.1579	27	-0.0409	0.8213	33	-0.0977	0.5138	47	0.0199	0.8780	62
**Blood Glucose***	-0.4325	0.0856	17	-0.1940	0.4524	17	0.1031	0.6240	25	-0.0099	0.9627	25
**Plasma Bicarbonate**	-0.4462	0.0004	60	-0.1513	0.2145	69	-0.5154	<0.0001	99	-0.2989	0.0023	102
**Plasma Bicarbonate ***	-0.2261	0.1849	36	-0.1883	0.2510	39	-0.6020	<0.0001	42	-0.2192	0.1631	42
**Respiration Rate**	0.141	0.1067	132	0.2043	0.0059	180	0.3123	<0.0001	178	0.1578	0.0150	237
**Respiration Rate***	-0.0048	0.9646	88	0.0634	0.5459	93	0.4683	<0.0001	67	0.0389	0.7544	67
**PO2**	-0.2046	0.1137	61	0.1441	0.2375	69	-0.0342	0.7378	98	-0.0948	0.3456	101
**PO2***	-0.2137	0.2040	37	0.0283	0.8644	39	-0.0248	0.8761	42	-0.0092	0.9537	42
**Temperature at enrolment**	-0.1519	0.0467	172	-0.2081	<0.0001	1213	-0.1006	0.1619	195	-0.2583	<0.0001	478
**Temperature at enrolment***	-0.0811	0.4251	99	-0.2900	<0.0001	272	-0.1146	0.3378	0.338	-0.2951	0.0017	111
**Blood Urea Nitrogen**	0.5458	<0.0001	125	0.1647	0.0289	176	0.6859	<0.0001	180	0.4660	<0.0001	241
**Blood Urea Nitrogen***	0.5381	<0.0001	82	0.2000	0.0649	86	0.7234	<0.0001	69	0.4391	0.0002	69
**Serum Creatinine**	0.4362	<0.0001	127	0.1964	0.0086	178	0.5855	0.5855	179	0.4705	<0.0001	241
**Serum Creatinine***	0.3939	0.0002	84	0.1803	0.0928	88	0.5633	<0.0001	69	0.4141	0.0004	69
**Bilirubin**	0.4594	<0.0001	119	0.1925	0.0147	160	0.6130	<0.0001	164	0.3727	<0.0001	214
**Bilirubin***	0.4491	<0.0001	78	0.1392	0.2153	81	0.6230	<0.0001	60	0.4113	0.0011	60
**Degree of severity**				0.7093	<0.0001	1215				0.7514	<0.0001	481
**Days in hospital**	0.4913	<0.0001	174				0.5999	<0.0001	197			

Supplemental Table 1: Univariate correlation of parasitological and clinical factors with degree of severity and length of hospitalization. Two datasets are shown here. The parameters with * represent the subset of samples whose parasite biomass was assessed. The parameters without * represent the whole dataset.
